# Early outcomes of drug-coated balloon angioplasty and stent placement for the treatment of iliac artery lesions

**DOI:** 10.3389/fsurg.2025.1598354

**Published:** 2025-07-03

**Authors:** Jia-Hao Wen, Chun-Min Li, Zhen-Yi Jin, Sheng-Xing Wang

**Affiliations:** The Department of Vascular Surgery, Beijing Chaoyang Hospital, Capital Medical University, Beijing, China

**Keywords:** atherosclerosis obliterans, iliac artery, drug-coated balloon, stent, endovascular therapy

## Abstract

**Objective:**

The efficacy and safety of drug-coated balloons (DCBs) in the treatment of aortoiliac artery stenosis or occlusion remains poorly explored.

**Methods:**

A single-center retrospective cohort study of patients diagnosed with iliac artery stenosis or occlusion who received either iliac artery DCB angioplasty or stent implantation was conducted at our institution. The patients were followed up 6 and 12 months postoperatively. Lower limb computed tomography angiography was performed during the follow-up period. The primary endpoint of the study was the primary patency at 6 and 12 months. Both the clinical and follow-up data were analyzed.

**Results:**

50 patients underwent DCB angioplasty, while 71 received stent implantation. Demographic and lesion characteristics were comparable between the two groups (*P* > 0.05). However, the balloon diameter used in the DCB group was significantly smaller (6.42 ± 0.80 mm vs. 7.39 ± 0.97 mm, *P* < 0.001). The primary patency values 6 and 12 months postoperatively were 84.2% and 80.7% for the DCB group and 96.1% and 89.6% for the stent group, respectively with no significant difference between the two groups (*P* = 0.124). However, the 12-month patency in the common iliac artery segment for the DCB group was significantly lower than that for the stent group (75.0% vs. 97.3%, *P* = 0.006). Univariate and multivariate logistic analyses did not identify any factors associated with long-term patency.

**Conclusion:**

Same as stents, DCBs maintained a favorable but lower patency rate across various calcification levels and different TASC Ⅱ classification in patients with aortoiliac artery stenosis or occlusion.

## Introduction

The aortoiliac arterial segment is a common site of involvement in lower extremity atherosclerotic diseases, with over a third of affected patients exhibiting stenotic or occlusive lesions (1, 2). The Inter-Society Consensus Document for the Management of Peripheral Arterial Disease [Trans-Atlantic Inter-Society Consensus (TASC)Ⅱ] classified aortoiliac artery disease into four anatomical types, initially recommending endovascular treatment for Type A and B lesions. Subsequently, endovascular treatment was favored for all lesion types in the 2015 update (3, 4). Stent implantation in the iliac artery is the preferred treatment modality for aortoiliac artery disease. A variety of stent types are extensively utilized in clinical practice, demonstrating high technical success rates and promising long-term patency rates, thus establishing themselves as the frontline therapy method (5–9).

Drug-coated balloons (DCBs) have become a prevalent treatment for femoropopliteal artery disease, leveraging chemotherapeutic agents to inhibit vascular endothelial proliferation in order to reduce restenosis. Their safety and efficacy have been well-documented in clinical trials (10–12). The use of DCBs for the treatment of restenosis lesions has been reported in aortoiliac artery disease (13, 14). However, they are infrequently recommended as a primary treatment option. Despite a general lack of prioritization for DCB use in aortoiliac artery disease in the existing guidelines, these recommendations are largely derived from expert opinion (15). Therefore, a retrospective analysis was conducted to evaluate the safety and efficacy of DCBs and to compare them with stents in the treatment of aortoiliac artery stenosis or occlusion.

## Methods

### Study design

A single-center retrospective cohort study was conducted between January 2019 and January 2023. Patients with iliac artery stenosis or occlusion who underwent either DCB (Orchid/Dahlia, Acotec Scientific, Beijing, China; paclitaxel: 3 µg/mm^2^) angioplasty (DCB group) or bare metal stent (Boston Scientific, Marlborough, Massachusetts) implantation (stent group) were enrolled. The surgeons selected the modus operandi according to the lesion characteristics and obtained informed consent. Follow-up assessments were scheduled at 6 and 12 months post-operation.

The study was exempt from the requirement for informed consent as it included no data that allowed patient identification and was approved by the Research Ethics Committee of the institution (2023-ke-695).

The criteria for surgery were as follows: age of ≥18 years; symptoms, such as intermittent claudication, ischemic resting pain, ulceration, or limb loss (Rutherford category 2–6); and computed tomography angiography (CTA)-confirmed severe stenosis and/or occlusion in the common iliac artery (CIA) and/or external iliac artery (EIA). Exclusion criteria were as follows: life expectancy of <1 year; prior open surgery or interventional therapy on the target lesions; presence of iliac aneurysm, acute arterial thrombosis, known allergy to stent graft or balloons coated with pharmaceuticals; and uncorrected coagulation dysfunction.

All procedures were conducted under local anesthesia. Access to the femoral artery or left brachial artery was obtained. A 6F catheter was utilized to access the target lesion via an ipsilateral retrograde or contralateral anterograde approach. Post-catheterization, patients were administered heparin at a dose of 80–100 IU/kg of body weight. A guide wire was used to navigate through the target lesion, followed by angiography to ascertain the lesion's characteristics, including location, length, diameter, and degree of calcification. Appropriately-sized and lengthened balloons or stents were selected based on the target vessel dimensions, with the stipulation that the device must extend at least 3 mm proximal and distal to the lesion (16). Lesion pre-dilation was performed using ordinary balloons with a diameter of <1 mm prior to balloon dilation or stent implantation. Balloon expansion duration was 2 min in the DCB group. An overlap of at least 5 mm between the balloons was maintained for cases requiring multiple DCBs. A stent was deployed if there was residual stenosis (>30%) or flow-limiting dissection after expansion. Balloon dilation was performed following stent implantation for patients in the stent group. An overlap of at least 1 cm was ensured when multiple stents were necessary (17). Patients presenting with stenosis or occlusion of the ipsilateral superficial femoral artery or distal outflow tract were also treated at the corresponding sites. All patients were prescribed clopidogrel (75 mg/day) and aspirin (100 mg/day) for at least 1 month after surgery and received aspirin (100 mg/day) indefinitely.

Clinical data for the study patients were collected, including demographic data [gender, age, body mass index (BMI), smoking history and drinking history], comorbidities (hypertension, diabetes mellitus, coronary artery disease, cerebrovascular disease, and chronic kidney disease), disease-specific characteristics [ankle brachial index (ABI), Rutherford grade, calcification, outflow tract condition, and TASC Ⅱ classification], surgical data, and postoperative related complications (flow-limiting dissection, distal embolization, bleeding, and infection).

All patients underwent postoperative angiography to assess the efficacy of the surgical intervention. Patients’ Rutherford classification, ABI and any adverse events were documented at 6- and 12-month follow-up visits which was routinely conducted in our medical center after discharge. In addition, all patients underwent a CTA procedure to assess target lesions. Prompt CTA was performed in patients exhibiting recurrent clinical symptoms during the follow-up period.

### Definitions

Primary patency, defined as freedom from severe stenosis or blood flow loss necessitating re-intervention, served as the primary endpoint of the study. Secondary patency was defined as freedom from stenosis regardless of several interventions. The Stenosis rate was determined using CTA by the ratio of the diameter of the narrowest to the nearby normal artery. Severe restenosis was diagnosed with a vascular stenosis rate of >70%. Calcification burden severity was assessed based on the Peripheral Arterial Calcium Scoring Scale (18) as follows: Grade 0, no visible calcification at the target lesion site; grade 1, unilateral wall calcification of <5 cm; grade 2, unilateral wall calcification of ≥5 cm; grade 3, bilateral wall calcification of <5 cm; and grade 4, bilateral wall calcification of ≥5 cm. Severe calcification was categorized as grade 3 or higher. Six grades of dissection (A–F) were identified. Type A was defined as dissection with minor radiolucent areas, type B as linear dissection, type C as dissection with contrast agent outside the lumen, type D as spiral dissection, type E as persistent filling defects, and type F as vessel occlusion without distal antegrade flow. Severe vessel dissection patterns were defined as type C or higher (19). Outflow tract patency was defined as femoral artery stenosis of <70% and infrapopliteal arteries stenosis of <50% without high-risk lesion features, such as thrombus or severe calcification.

### Sample calculation

This investigation was designed as a non-inferiority trial. The stent group demonstrated a 12-month primary patency rate of approximately 90%. Given the limited existing evidence regarding DCB applications for aortoiliac arterial disease, previous investigations have reported an approximate 80% primary patency rate for DCB treatment of aortoiliac restenosis. Consequently, we hypothesized an 80% primary patency rate for the DCB cohort in the present study. Sample size determination using a 10% non-inferiority margin, a unilateral significance level of 0.025, and 80% statistical power indicated a requirement of 50 participants per group, yielding a total required sample size of 100 participants.

### Statistical analysis

Continuous variables were expressed as mean ± standard deviation. Categorical variables were expressed as frequencies and percentages. The *χ*^2^ test and t test were used for analysis of categorical and continuous data, respectively. The patency rates at 6 and 12 months were estimated using the Kaplan–Meier life table. Univariate and multivariate Cox proportional hazards models were used to identify factors associated with primary patency. *P* value of <0.05 was considered statistically significant. All data were analyzed using SPSS (IBM SPSS Statistics version 26).

## Results

### Baseline characteristics

Among the 121 patients enrolled in the study, 50 were treated with DCBs and 71 with stents. Demographic characteristics, including gender, age, and BMI, were comparable between the two groups. Furthermore, there were no significant differences in the prevalence of comorbidities, smoking history, and drinking history. Preoperative assessments, such as ABI, Rutherford category, and TASC Ⅱ classification, were also similar between the DCB and stent groups (Table 1). The results of normality tests for continuous variables were shown in Supplementary Table S1.

**Table 1:** Baseline characteristics.

Baseline characteristics	DCB [*N* (%)/mean ± SD]	STENT [*N* (%)/mean ± SD]	*t*-value/chi-square	*P*-value
Male	43 (86.0)	68 (95.8)	0.011	0.112
Age, years	67.44 ± 9.31	67.38 ± 8.07	−0.415	0.212
BMI	24.22 ± 3.23	23.74 ± 3.04	0.662	0.509
Hypertension	38 (76.0)	47 (66.2)	0.992	0.245
DM	21 (42.0)	23 (32.4)	0.802	0.279
CAD	17 (34.0)	19 (26.8)	0.183	0.391
CVD	13 (26.0)	10 (14.1)	2.984	0.100
CKD	4 (8.0)	2 (2.8)	1.429	0.385
Smoking	26 (52.0)	45 (63.4)	1.044	0.211
Drinking	16 (32.0)	25 (35.2)	0.051	0.713
ABI	0.64 ± 0.22	0.63 ± 0.19	0.427	0.810
TASC Ⅱ			1.268	0.763
A	19 (38.0)	24 (33.8)		
B	13 (26.0)	19 (26.8)		
C	7 (14.0)	7 (9.9)		
D	11 (22.0)	21 (29.6)		
Rutherford category			2.343	0.271
2	14 (28.0)	14 (19.7)		
3	26 (52.0)	42 (59.2)		
4	9 (18.0)	11 (15.5)		
5	0	1(1.4)		
6	1(2.0)	3(4.2)		

BMI, body mass index; DM, diabetes mellitus; CAD, coronary artery disease; CVD, cerebrovascular disease; CKD, chronic kidney disease; ABI, ankle brachial index; TASC, trans-atlantic inter-society consensus.

### Procedure data

A total of 57 limbs were treated with DCBs, while 80 limbs received a stent implantation. There were no significant differences in lesion location, calcification degree, outflow tract condition, lesion length, or stenosis degree of the target lesions between the two groups. However, the balloon diameter used in the DCB group was significantly smaller (6.42 ± 0.80 mm vs. 7.39 ± 0.97 mm, *P* < 0.001, Table 2).

**Table 2:** Lesion characteristics.

Lesion characteristics	DCB [*N* (%)/mean ± SD]	STENT [*N* (%)/mean ± SD]	*t*-value/chi-square	*P*-value
Anatomic location			0.470	0.790
CIA	24 (42.1)	37 (46.3)		
EIA	21 (36.8)	25 (33.6)		
CIA & EIA	12 (21.1)	18 (22.5)		
Severe calcification	30 (61.4)	31 (38.8)	2.579	0.084
Poor outflow tract	25 (43.9)	34 (42.5)	0.025	0.874
Lesion length, cm	4.95 ± 4.05	4.97 ± 3.63	−0.007	0.969
Device diameter, mm	6.42 ± 0.80	7.39 ± 0.97	−5.588	<0.001

DCB, drug coated balloon; CIA, common iliac artery; EIA, external iliac artery.

During the procedure, there were no complications occurred such as dissection, artery rupture or remote embolization. However, minor bleeding at the puncture site occurred in one patient in the DCB group. This was managed with local compression and did not require further intervention. Two patients in the DCB group had a non-flow-limiting dissection. The stent group experienced no other complications.

### Follow-up

**Figure 1:**
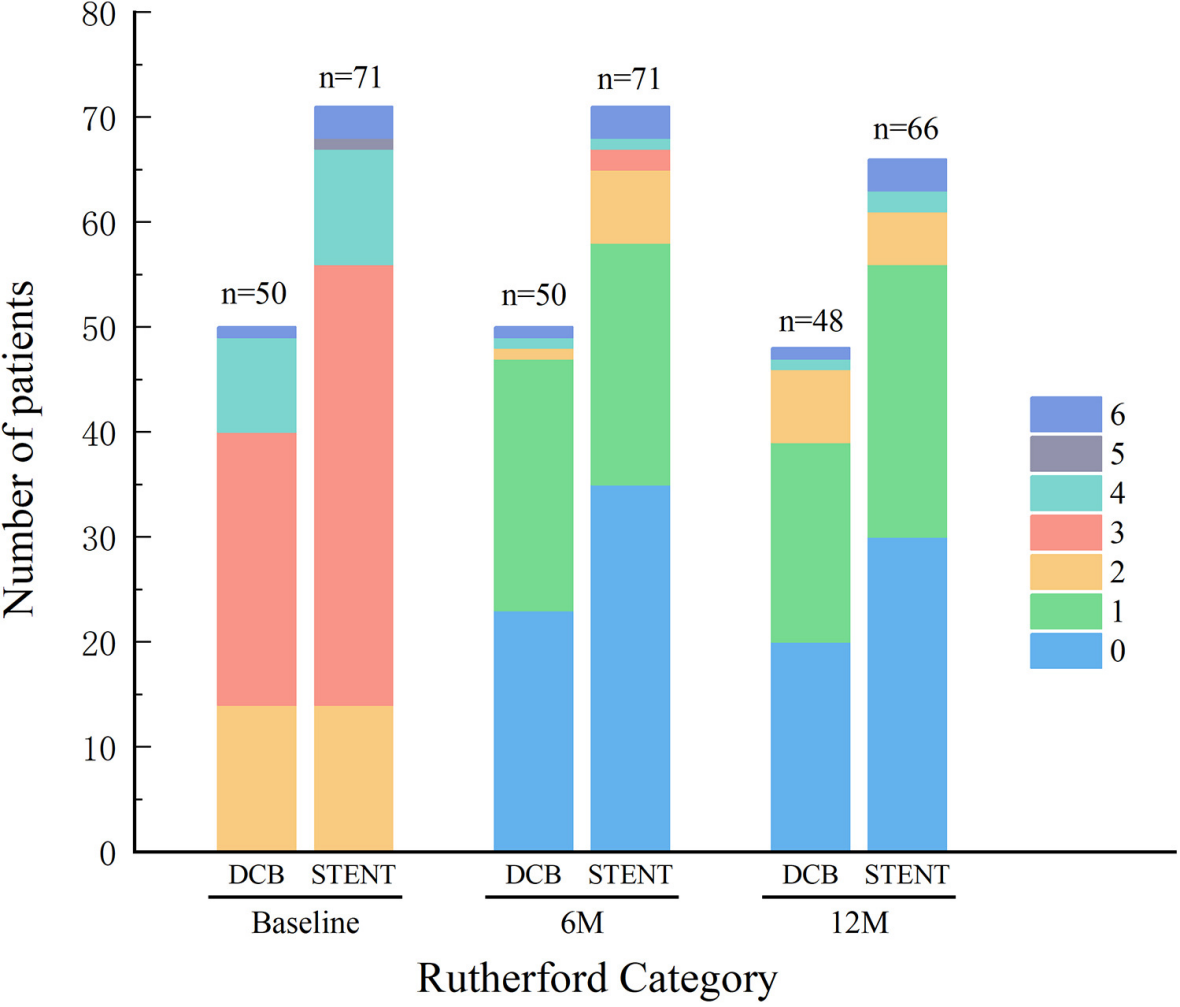
Rutherford category. Preoperative and postoperative Rutherford category was similar between the drug-coated balloon (DCB) group and stent group (STENT), with no significant differences observed. And the Rutherford category for both groups demonstrated significant improvement at the 6-month follow-up (6M) and the 12-month follow-up (12M).

**Figure 2:**
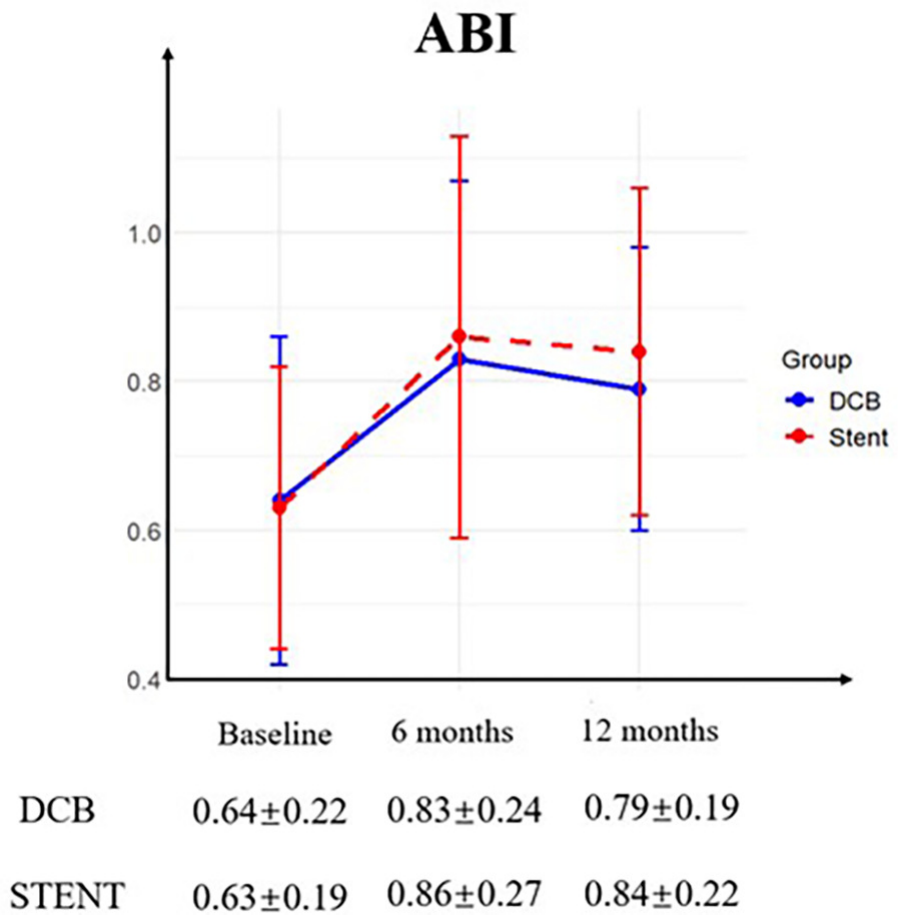
Ankle-brachial index (ABI). Preoperative and postoperative ABI was similar between the drug-coated balloon (DCB) group and stent group (STENT), with no significant differences observed. And the ABI for both groups demonstrated significant improvement at the 6-month follow-up and the 12-month follow-up.

All patients successfully completed the 6-month postoperative follow-up. However, two patients in the DCB group were lost to follow-up by the 12-month timepoint. Four patients were lost to follow-up at 12 months and one patient expired due to chronic kidney disease (CKD) within the stent group. The Rutherford category for both groups demonstrated significant improvement at both follow-up timepoints, with no significant differences observed between the DCB and stent groups (Figure 1). Furthermore, during the follow up, the ABI in both groups has greatly improved without differences between the groups (Figure 2).

### Patency

**Figure 3:**
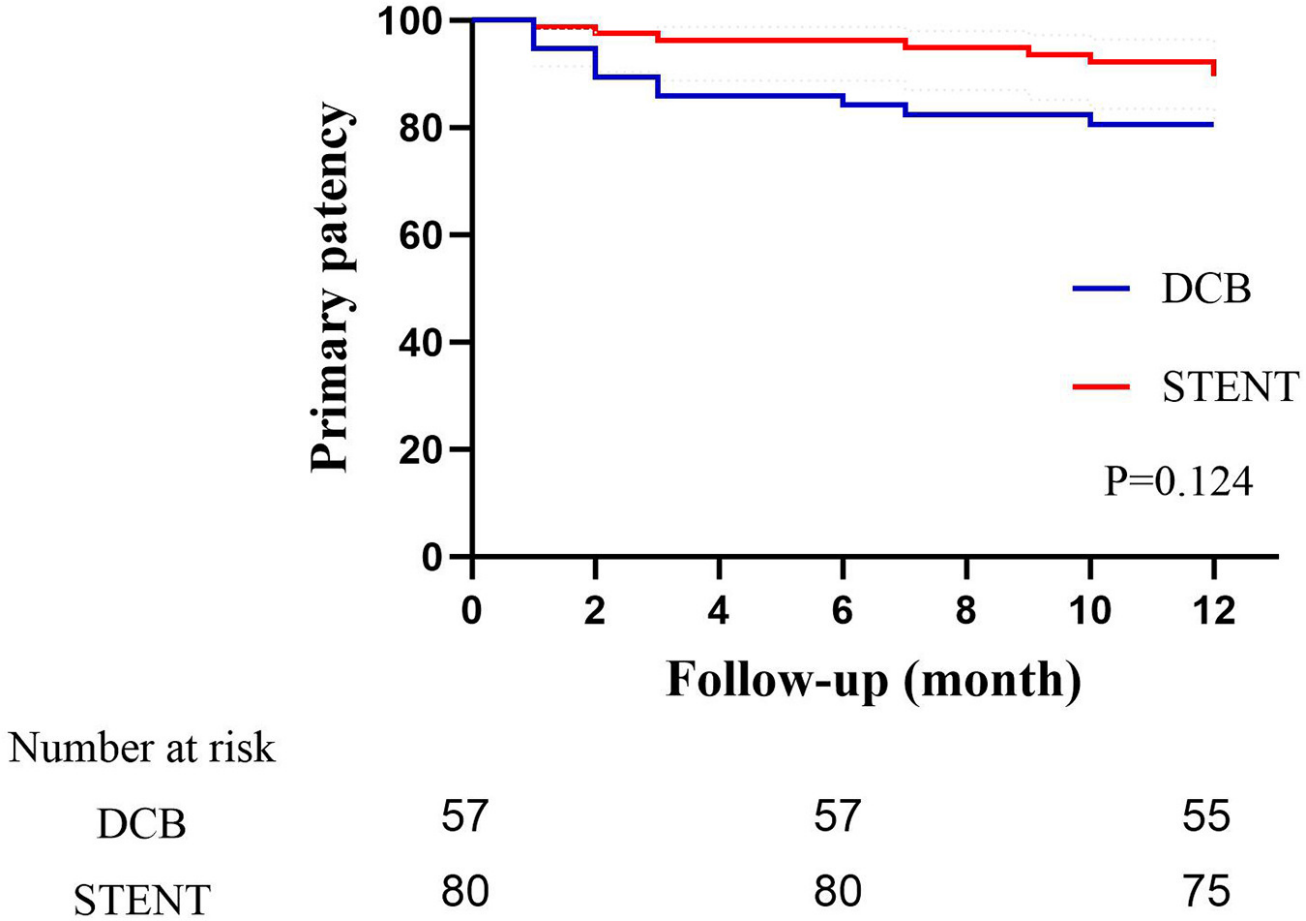
Kaplan–Meier estimate of primary patency on follow-up angiography between drug-coated balloon group (DCB) and stent group (STENT). Although the stent group exhibited a higher patency rate, there was no significant difference (*P* = 0.124).

**Figure 4:**
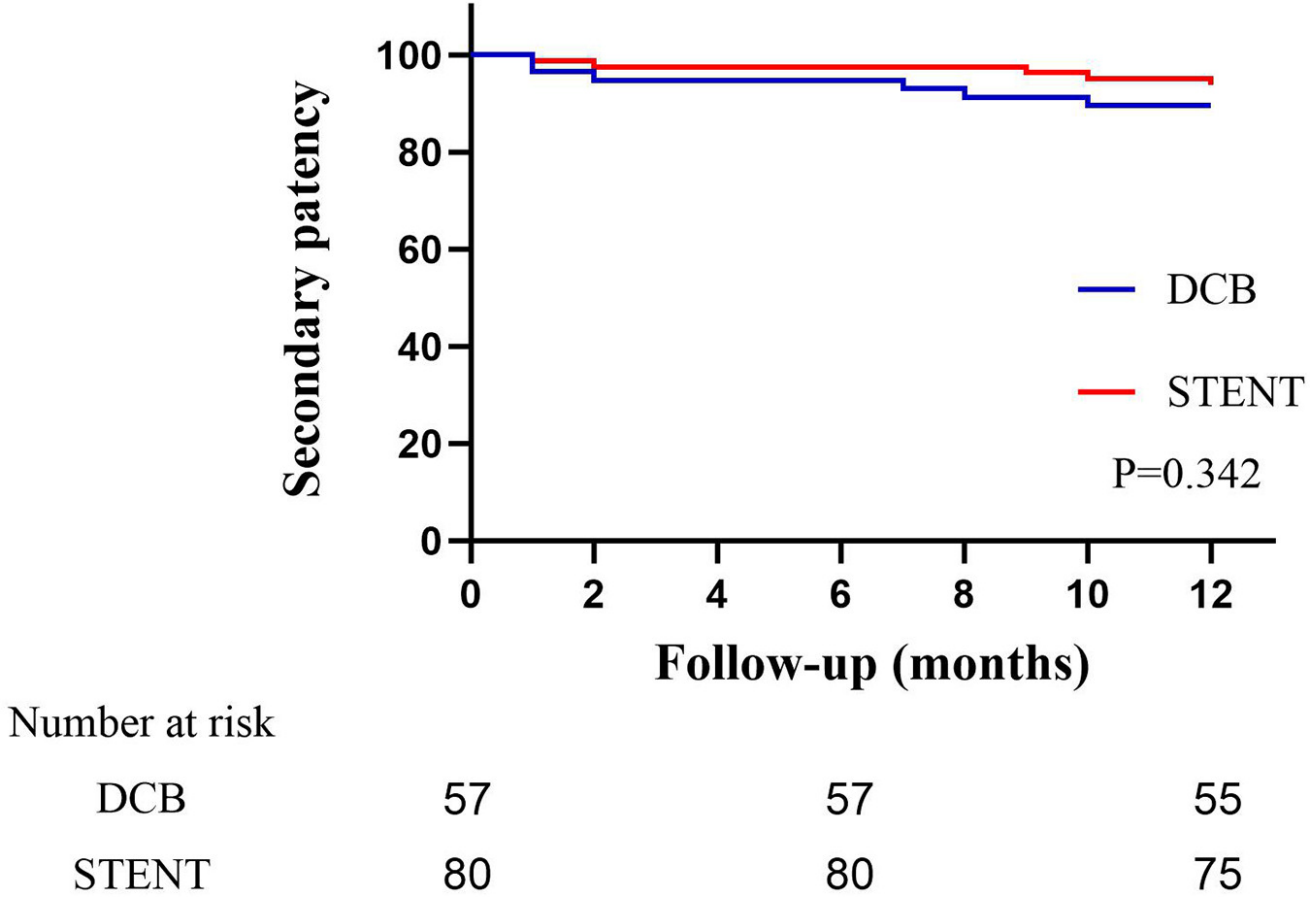
Kaplan–Meier estimate of secondary patency on follow-up angiography between drug-coated balloon group (DCB) and stent group (STENT). Although the stent group exhibited a higher patency rate, there was no significant difference (*P* = 0.342).

**Figure 5:**
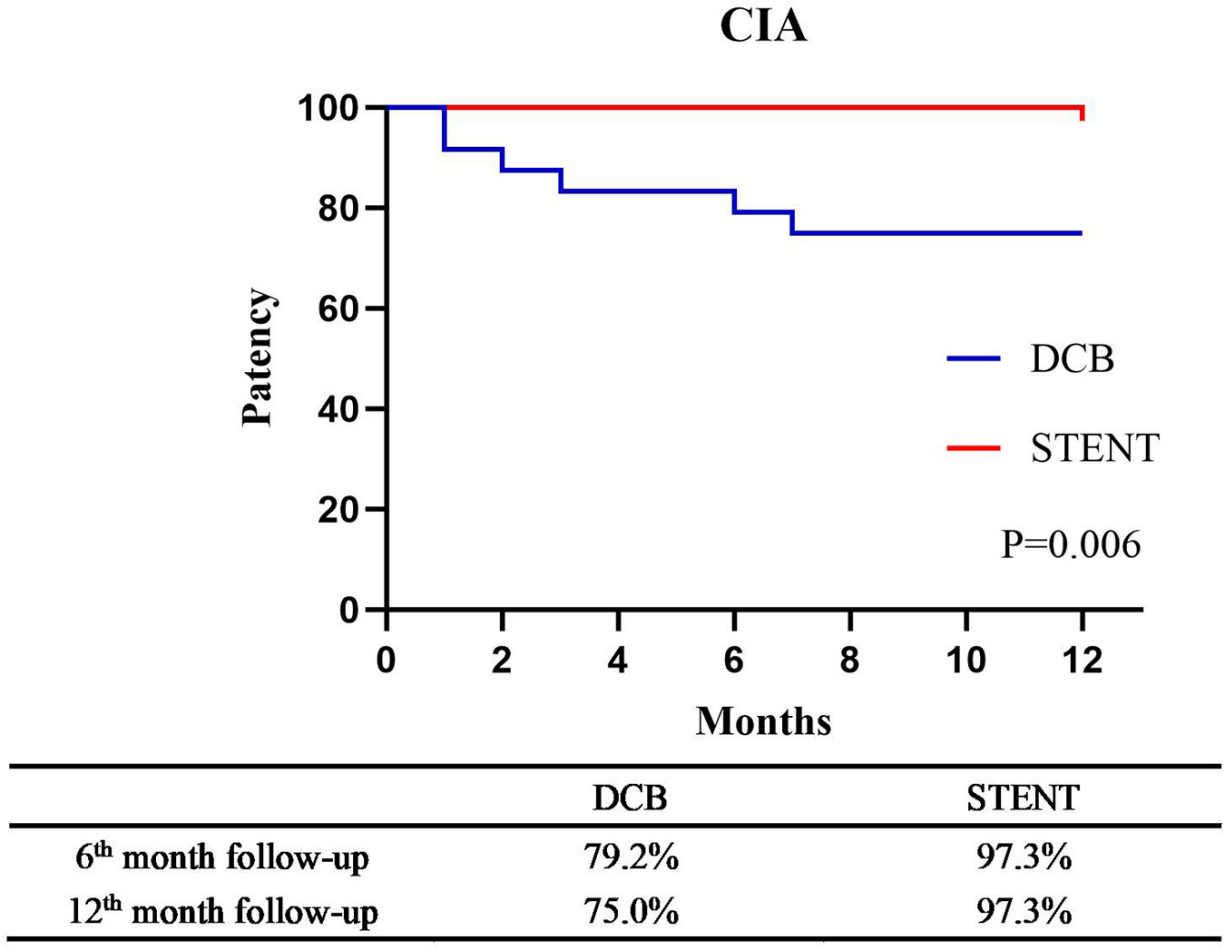
Kaplan–Meier estimate of primary patency for the common iliac artery (CIA) segement between drug-coated balloon group (DCB) and stent group (STENT) at 6-month follow-up and the 12-month follow-up (*P* = 0.006).

The primary patency rates for the DCB group at 6 and 12 months were 84.2% (95% CI: 74.79%–93.61%） and 80.7% (95% CI: 70.50%–90.89%), and the primary patency rates for the stent group were 96.1% (95% CI: 91.79%–100.41%) and 89.6%(95% CI: 82.74%–96.46%) (Figure 3, *P* = 0.124). The secondary patency rates for the DCB group at 6 and 12 months were 94.7% (95% CI: 88.82%–100.58%) and 89.5% (95% CI: 81.46%–97.53%), and the secondary patency rates for the stent group were 97.4% (95% CI: 93.87%–100.93%) and 93.5% (95% CI: 88.01%–98.99%) respectively (Figure 4, *P* = 0.342). Although the stent group exhibited a higher primary and secondary patency rates, there was no significant difference between the two groups. However, the 12-month patency rate in the CIA segment for the DCB group was significantly lower than that for the stent group [75.0% (95% CI: 57.75%–92.25%) vs. 97.3% (95% CI: 91.98%–102.49%), *P* = 0.006, Figure 5]. In contrast, this difference was not statistically significant in the EIA [85.7% (95% CI: 70.84–100.59%) vs. 83.6% (95% CI: 68.70%–98.49%), *P* = 0.874] or the CIA and EIA [83.3% (95% CI: 62.13%–104.47%) vs. 80.0% (95% CI: 61.97%–98.037%), *P* = 0.870] segment.

In addition, subgroup analyses were conducted for the TASC A and B as well as C and D lesions. In the TASC A and B group, the 12-month patency for the DCB group was 86.1%, compared to 93.2% for the stent group. However, this difference was not statistically significant (*P* = 0.241). A similar trend was observed in the TASC C and D subgroup, with patency rates of 70.8% for the DCB group and 84.7% for the stent group (*P* = 0.228). Furthermore, there were no significant differences in patency rates across different lesion types within both the DCB and stent groups (*P* = 0.216 and *P* = 0.168, respectively).

No significant differences were observed when analyzing the 12-month patency rates for the DCB and stent groups across varying degrees of calcification. In cases of non-severe calcification, the patency rates were 86.4% for the DCB group and 95.9% for the stent group (*P* = 0.143). Similarly, the rates were 76.9% and 78.2% for severe calcification in the DCB and stent groups, respectively (*P* = 0.724).

### Risk factor analysis

After conducting a univariate analysis related to primary patency, factors with *P* < 0.05 were selected for multivariate analysis. The degree of calcification, balloon or stent diameter, hypertension, and history of cerebrovascular disease were included (Table 3). Multivariate analysis did not identify any factors related to patency rate (Table 3).

**Table 3:** Factors associated with primary patency.

Factors	Univariate analysis		Multivariate analysis			
	Test statistics	*P*-value	Test statistics	*P*-value	HR	95% CI
Operation program	2.587	0.108				
Anatomic location	0.646	0.724				
Severe calcification	7.613	0.017	3.469	0.063	0.390	0.135–1.123
Lesion length^a^	2.586	0.275				
Stenosis degree^b^	0.355	0.551				
Poor outflow tract	2.264	0.132				
Device diameter^c^	6.940	0.008	2.663	0.103	2.678	0.738–9.718
TASC Ⅱ (A + B vs. C + D)	3.022	0.083	4.497	0.221	0.546	0.216–1.376
Rutherford category^d^	1.148	0.887				
Sexuality	0.104	0.748				
Age^e^	0.725	0.395				
BMI^f^	1.477	0.224				
Hypertension	3.758	0.053	3.063	0.080	0.269	0.062–1.170
DM	0.100	0.751				
CAD	0.020	0.888				
CVD	4.502	0.034	0.001	0.972	698543.384	0.000-
CKD	1.068	0.301				
Smoking	0.230	0.631				
Drinking	1.009	0.315				

The grey shaded part indicates that after univariate analysis, these factors are included in the multivariate analysis.

TASC, trans-atlantic inter-society consensus; BMI, body mass index; DM, diabetes mellitus; CAD, coronary artery disease; CVD, cerebrovascular disease; CKD, chronic kidney disease; HR, hazard ratio; CI, confidence intervals.

^a^ Lesion length was divided into: ≤3 cm, 3–10 cm, >10 cm.

^b^ Stenosis degree was divided into: stenosis, occlusion.

^c^ Device diameter was divided into: <8 cm, ≥8 cm.

^d^ Rutherford category was divided into: >4, ≤4.

^e^ Age was divided into: <70 years old, ≥70 years old.

^f^ BMI was divided into: <24, ≥24.

## Discussion

Iliac artery stenosis or occlusion represents a common disease in atherosclerosis obliterans of lower limb. Endovascular stent implantation is the preferred method for its treatment (15). In the present study, the respective 6- and 12-month patency values for the stent group were 96.1% and 89.6%, which were consistent with those observed in previous studies (the 12-month patency rate was 84.0%–94.0%) (6, 7, 14, 20). The 6- (84.2%) and 12-month (80.7%) patency rates of the DCB group did not demonstrate statistically significant differences when compared to the stent group. However, the overall patency rate for the DCB group was lower. This disparity was most pronounced in the CIA segment, where a statistically significant difference was observed (75.0% for DCB vs. 97.3% for stent, *P* = 0.006).

First, the degree of calcification in the DCB group was higher compared to that in the stent group although there was no statistically significance, which introduced greater surgical complexity and potentially impacted medium- to long-term outcomes (9, 21). This may also account for the observed lower patency rates in the DCB group. However, despite a higher degree of calcification in the DCB group, this parameter was not significantly associated with long-term patency rates, which was consistent with previous study results (16, 22). In a study by Bekke et al., moderate to severe calcification predicted a lower restenosis rate [*P* = 0.021, hazard ratio (HR) = 0.3] (23). This discrepancy may be attributed to the fact that more heavily calcified vessels exhibited poorer vascular endothelial activity, reducing the likelihood of endothelial proliferation. Further research is still needed to explore the relationship between calcification and long-term patency.

Second, the mean diameter of the devices utilized in the DCB group was significantly lower compared to that in the stent group. This could be attributed to the use of a smaller balloon size during the operation due to the safety consideration resulting in a reduced DCB diameter and consequently leading to a smaller post-operative diameter of the blood vessels in the DCB group. Squizzato et al. demonstrated that stent diameters of ≥8 cm were effective in preventing occlusion and restenosis (HR = 2.86, *P* = 0.03), while stent diameters of <8 mm were associated with poorer long-term patency (HR = 8.5, *P* < 0.001) (9, 24). Moreover, Piazza et al. confirmed that smaller vessels were not conducive to long-term patency of iliac artery, especially when the stent diameter was <7 cm (HR = 2.86, *P* = 0.01) (25). Notably, the lower average diameter of the devices used in the DCB group could have influenced the patency.

Overall, there was no statistically significant difference in the long-term patency between the two groups, which may be due to the small sample size and inability to detect the difference. Furthermore, previous studies have not demonstrated superior performance compared to conventional balloons in the treatment of iliac artery restenosis (14), suggesting that further research is necessary to elucidate the role of DCBs in the CIA segment. On the contrary, the present study observed no difference in the 12-month patency rates in the EIA segment between the two groups, with the DCB group showing a slightly higher rate (85.7% vs. 83.6%, *P* = 0.874). Due to limited sample size in the present study, further research is needed to verify this result. Furthermore, it should be pointed out that good adaptability was required for the selection of stents deployed in the EIA in order to reduce the risk of stent collapse or fracture during exercise (9, 26). At the same time, calcification severity and stent length were recognized as risk factors for stent fracture (27–29). Notably, DCB angioplasty did not include graft implantation, thereby eliminating the risk of stent fracture. Thus, the use of DCBs may become another option for EIA endovascular treatment in the future.

As mentioned earlier, calcification can impact the long-term prognosis in patients undergoing endovascular treatment of the iliac artery. The present study results revealed that both DCBs and stents had lower long-term patency rates in severely calcified lesions. The more severe the calcification of the vessel, the more likely it is to establish restenosis and be affected by impeded drug delivery and retention. Therefore, auxiliary techniques are needed to reduce the degree of calcification in severely calcified lesions and improve patient prognosis (30). Stavroulakis et al. demonstrated that the combination of intravascular lithotripsy (IVL) and DCBs was effective for severe calcification in the common femoral artery, with minimal complications and satisfactory patency rates (31). Adams et al. and Radaideh et al. also reported good long-term patency rates when using IVL combined with balloon-based technologies in the iliac artery and other lower limb arteries (32, 33). Furthermore, plaque rotational resection combined with DCBs or IVL combined with DCBs has been shown to effectively address stenosis in patients with restenosis lesions (34–36). In summary, the combination of plaque debulking with DCBs or stents may offer better outcomes for iliac artery stenosis or occlusion lesions.

The present study had several limitations. First, it was a retrospective, small-scale, non-randomized study, and the results need to be confirmed in larger studies or randomized controlled trials. And because “randomization” was not feasible in retrospective observational studies, although the allocation was determined by the surgeon, matching or hierarchical analysis and other steps were not feasible, which may further limited the effectiveness of the results. In addition, the study's small sample size may have prevented the detection of some uncommon clinical comorbidities and events. For the patients who were lost to follow-up, we used the last observation carried forward processing, ignored the dynamic changes of the data, and underestimated the data variability and statistical error, resulting in biased results. Although there were no deaths attributed to chemotherapy drugs, the long-term survival rate of patients in the DCB group remains unknown due to the short follow-up time. Continued follow-up with the patients will allow to determine the long-term survival rates.

## Conclusion

Same as stents, DCBs maintained a favorable patency rate across various calcification levels and different TASC Ⅱ classification in patients with aortoiliac artery stenosis or occlusion. Although in this research, there were no statistical significance compared with stents, DCBs have a low priority. Besides, the suitability of DCBs for treatment of common iliac artery lesions remains uncertain. The long-term benefits of DCBs in other arterial segments require further investigation.

## Data Availability

The raw data supporting the conclusions of this article will be made available by the authors, without undue reservation.
