# Supplementary material for: Early outcomes of drug-coated balloon angioplasty and stent placement for the treatment of iliac artery lesions
Source: Front Surg. 2025 Jul 3;12:1598354. doi: 10.3389/fsurg.2025.1598354 (PMC12267272; doi:10.3389/fsurg.2025.1598354)
Supplement: Supplementary file 1 [file Table1.docx]

**Supplementary Table 1. Normality tests**

|  | DCB | | STENT | |
| --- | --- | --- | --- | --- |
|  | Test statistics | P-value | Test statistics | P-value |
| Age | 0.116 | 0.056 | 0.086 | 0.175 |
| BMI | 0.136 | 0.011 | 0.063 | 0.200 |
| ABI | 0.079 | 0.162 | 0.081 | 0.134 |
| Lesion length, cm | 0.262 | 0.000 | 0.398 | 0.000 |
| Device diameter, mm | 0.294 | 0.000 | 0.243 | 0.000 |

BMI, body mass index; ABI, Ankle brachial index
