# Supplementary material for: Early outcomes of drug-coated balloon angioplasty and stent placement for the treatment of iliac artery lesions
Source: Front Surg. 2025 Jul 3;12:1598354. doi: 10.3389/fsurg.2025.1598354 (PMC12267272; doi:10.3389/fsurg.2025.1598354)
Supplement: Supplementary file 2 [file Table2.docx]

**Supplementary Table 2. Variance Inflation Factor (VIF)**

|  | Test statistics | Tolerance | VIF |
| --- | --- | --- | --- |
| Operation program | -0.881 | 0.531 | 1.882 |
| Anatomic location | -1.507 | 0.547 | 1.828 |
| Severe calcification | 1.323 | 0.738 | 1.354 |
| Lesion length ^a^ | 0.914 | 0.498 | 2.007 |
| Stenosis degree ^b^ | 0.081 | 0.685 | 1.459 |
| Poor outflow tract | 0.819 | 0.806 | 1.241 |
| Device diameter ^c^ | -0.969 | 0.526 | 1.902 |
| TASC Ⅱ (A+B vs. C+D) | 0.962 | 0.569 | 1.758 |
| Rutherford category ^d^ | 0.330 | 0.749 | 1.335 |
| Sexuality | -0.939 | 0.764 | 1.310 |
| Age ^e^ | -0.344 | 0.813 | 1.230 |
| BMI ^f^ | 1.256 | 0.789 | .268 |
| Hypertension | 2.109 | 0.810 | 1.235 |
| DM | -0.155 | 0.764 | 1.309 |
| CAD | 0.746 | 0.756 | 1.323 |
| CVD | -2.470 | 0.780 | 1.283 |
| CKD | -1.134 | 0.825 | 1.211 |
| Smoking | 0.477 | 0.572 | 1.747 |
| Drinking | -0.514 | 0.634 | 1.577 |

TASC, Trans-Atlantic Inter-Society Consensus; BMI, body mass index; DM, Diabetes mellitus; CAD, Coronary artery disease; CVD, Cerebrovascular disease; CKD, Chronic kidney disease

a．Lesion length was divided into: ≤3cm, 3-10cm, ＞10cm; b. Stenosis degree was divided into: stenosis, occlusion; c. Device diameter was divided into: ＜8cm, ≥8cm; d. Rutherford category was divided into: ＞4, ≤4; e. Age was divided into: ＜70 years old, ≥ 70 years old; f. BMI was divided into: ＜24, ≥24.
